# *Peltophorum pterocarpum* flower mediated synthesis of silver nanoparticles and its catalytic degradation of Acid Blue 113 dye

**DOI:** 10.1038/s41598-025-11980-1

**Published:** 2025-07-21

**Authors:** Ramesh Vinayagam, Stuthi A. Shetty, Gokulakrishnan Murugesan, Louella Concepta Goveas, Thivaharan Varadavenkatesan, Raja Selvaraj

**Affiliations:** 1 Department of Chemical Engineering, Manipal Institute of Technology, Manipal Academy of Higher Education, Manipal, Karnataka 576104 India; 2 Department of Biotechnology, M.S. Ramaiah Institute of Technology, Bengaluru, Karnataka 560054 India; 3 Department of Biotechnology Engineering, Nitte (Deemed to be University), NMAM Institute of Technology (NMAMIT), Nitte, India; 4 Department of Biotechnology, Manipal Institute of Technology, Manipal Academy of Higher Education, Manipal, Karnataka 576104 India

**Keywords:** Silver nanoparticles, *Peltophorum pterocarpum*, Acid Blue 113, Dye degradation, Green synthesis, Wastewater treatment, Pollution remediation, Green chemistry, Sustainability

## Abstract

In this study, silver nanoparticles (AgNPs) were synthesized using a green method involving *Peltophorum pterocarpum* flower extract. Successful formation of AgNPs was confirmed by a characteristic surface plasmon resonance peak at 416 nm in the UV–Vis spectrum. SEM and TEM analyses revealed uniformly spheroidal nanoparticles with an average size of 25.77 nm, while EDX confirmed the presence of silver. SAED patterns showed bright spots, indicating a polycrystalline nature, which was further supported by XRD, revealing a crystallite size of 15.58 nm and a lattice parameter of 0.4007 nm. FTIR spectra identified hydroxyl and carboxyl groups as key agents in nanoparticle reduction and stabilization. DLS analysis reported a hydrodynamic diameter of 99.41 nm and a PDI of 0.326, suggesting good monodispersity. The nanoparticles exhibited good stability with a zeta potential of − 14.7 mV. Catalytic studies showed rapid degradation of Acid Blue 113 dye in the presence of NaBH_4_, achieving a rate constant of 0.247 min^−1^ at 30 mg/L. These results demonstrate the environmental remediation potential of biogenically synthesized AgNPs and underscore the benefits of sustainable green synthesis approaches for industrial wastewater treatment.

## Introduction

Freshwater is indispensable for all forms of life, yet its purity is increasingly compromised by industrial activities. A significant contributor to this pollution is the dye industry, which uses dye extensively in textiles, paper, pharmaceuticals, food processing, and personal care products. India, as a major hub in Asia for dye production, faces substantial environmental challenges due to the effluent from textile printing and washing, which contains high volumes of dye pigments^1^. Such effluents pose serious threats to aquatic ecosystems and the broader environment, disrupting the food chain and potentially causing harm to human health through skin contact and accumulation in the food. Specific health risks linked to these contaminants include allergic reactions, neurological damage, and carcinogenic effects^2,3^.

Acid Blue 113 (AB 113), an anionic azo dye, is extensively employed to dye many products, constituting a significant fraction of global dye consumption^4^. Its pervasive use leads to the generation of substantial volumes of wastewater. The environmental and health ramifications of AB 113 are intensified by its chemical composition, which includes aromatic sulphonic groups renowned for their persistence and bioaccumulative nature^5^. Consequently, there is a pressing need for effective methods to eradicate AB 113 from wastewater. While adsorption techniques are commonly employed, they merely transfer the pollutant from the liquid to the solid phase without achieving complete removal^6^. In contrast, the degradation of dye-laden wastewater using nanoparticles presents a more effective solution, offering the potential for a complete breakdown of the dye molecules^7^.

Silver nanoparticles (AgNPs) are increasingly recognized for their exceptional physical, chemical, and mechanical properties, making them a promising solution for various applications^8^. Although there are many methods to synthesize AgNPs, including physical and chemical techniques, green synthesis stands out for its significant advantages. This eco-friendly approach is not only scalable and energy-efficient but also circumvents the need for high temperatures, pressures, or hazardous chemicals, aligning closely with sustainable practices and enhancing the biocompatibility of the nanoparticles^9^. Recent studies have highlighted the successful use of diverse plant extracts for AgNPs synthesis, including *Kalanchoe brasiliensis*^1^, *Acacia ehrenbergiana* cortex^10^, *Punica granatum* fruit peels^7^ and *Peltophorum pterocarpum* leaves^11^, showcasing their potential in catalytic dye degradation.

*Peltophorum pterocarpum*, commonly known as the “yellow flame tree,” is a tropical species belonging to the Fabaceae family. Its vibrant flowers are rich in a variety of phytochemicals, including carbohydrates, reducing sugars, sterols, tannins, glycosides, and flavonoids^12^. These bioactive compounds are known to play a crucial role in the green synthesis of nanoparticles, acting as both reducing and capping agents. The abundance of these natural constituents makes *P. pterocarpum* flower extract a promising and sustainable choice for the synthesis of AgNPs. Additionally, previous reports have demonstrated the antibacterial efficacy of AgNPs synthesized using this extract^13^, further validating its suitability. Motivated by these findings, the present study investigates a simple, eco-friendly method for synthesizing stable AgNPs utilizing *P. pterocarpum* flower extract in an aqueous silver nitrate medium.

The primary goal of the study is to investigate the potential of AgNPs synthesized using *P. pterocarpum* extract for catalyzing the degradation of AB 113 dye under the influence of sodium borohydride (NaBH_4_), offering an application in mitigating industrial dye pollution. Extensive characterization has been conducted on these AgNPs, synthesized through an eco-friendly green method, to confirm their morphology, stability, and functional properties, ensuring their efficacy in the targeted application.

## Experimental sections

### Materials and chemicals

Silver nitrate was sourced from Merck and Acid Blue 113 dye was obtained from Thomas Baker, Mumbai. Sodium borohydride was acquired from SRL. For all preparations, distilled water was used as the solvent, maintaining the purity of the solutions. Additionally, *P. pterocarpum* flowers were collected from the college campus.

### Synthesis of flower extract and AgNPs

The collected *P. pterocarpum* flowers were thoroughly cleansed with distilled water to eliminate any impurities and then dried. About 5 g of these cleaned flowers were dispersed in 50 mL of distilled water. This mixture was subsequently heated to 80 °C for one hour, during which the contents transitioned from colorless to pale yellowish color, indicating the extraction of bioactive compounds. After heating, the flower extract was cooled to ambient conditions, filtered to remove solid particles, and then stored at 4 °C in a refrigerator to preserve its chemical integrity for future use.

**Fig. 1:**
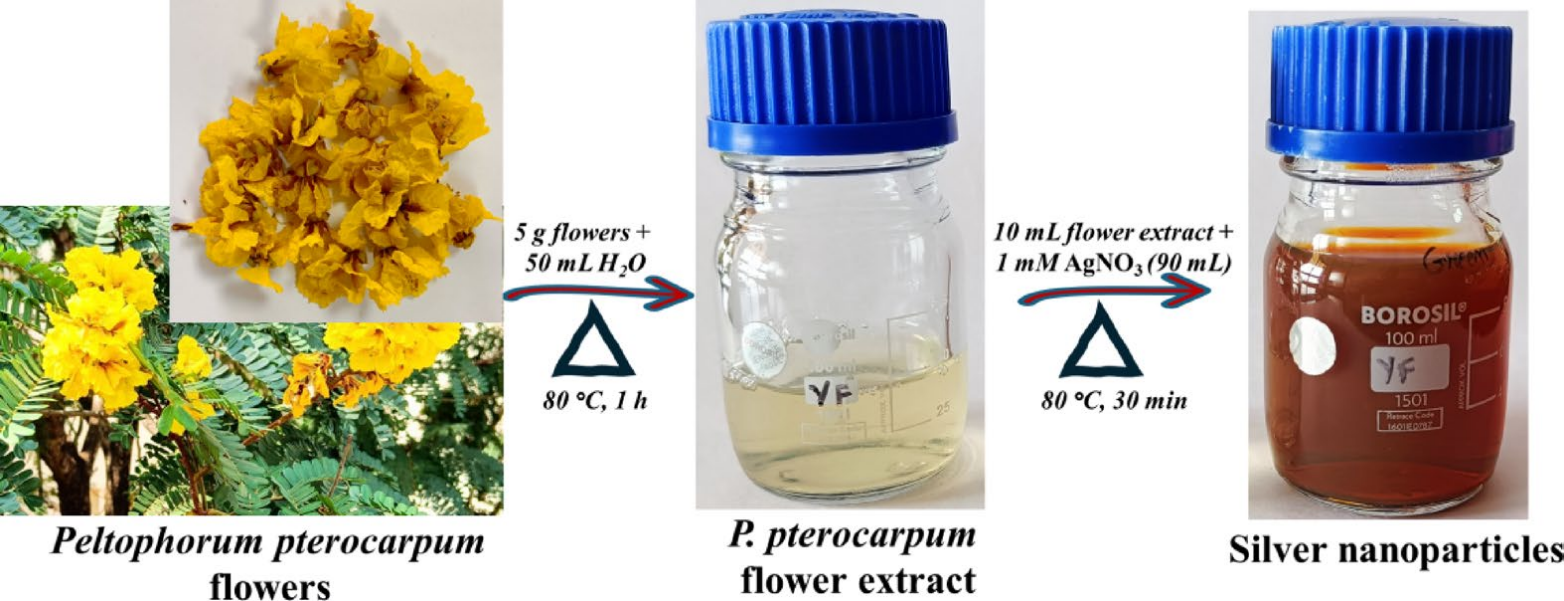
Schematic of green synthesis of silver nanoparticles using *Peltophorum pterocarpum* flower extract.

To synthesize AgNPs, initially, 10 mL of the prepared flower extract was heated to 80 °C and continuously stirred for 30 min to activate the phytochemicals responsible for the reduction. Subsequently, 90 mL silver nitrate (1 mM) was gradually added to the heated extract. This slow addition is crucial as it allows for controlled nucleation of the silver ions, which is essential for forming uniformly sized nanoparticles. The contents were then maintained at 80 °C for another hour to ensure complete nucleation and to stabilize the newly formed AgNPs. During this process, the solution underwent a noticeable color change to brown, a visual endorsement of the successful formation of AgNPs (Fig. 1). This color change is indicative of the surface plasmon resonance (SPR) phenomenon, typical of AgNPs, which occurs because of the collective oscillations of electrons of AgNPs induced by interaction with light^10^. The resulting colloidal solution, named as YFF-AgNPs, was then cooled to room temperature and stored for further characterization and analysis.

### Instrumentation

The synthesized YFF-AgNPs, were extensively characterized using a series of sophisticated analytical methods to ascertain the structural and functional attributes. UV-visible spectroscopy (UV-1900i, Shimadzu) provided the initial confirmation of nanoparticle synthesis through the observation of SPR. Further molecular insights were gained via Fourier Transform Infrared Spectroscopy (FTIR, Shimadzu-8400 S), which recognized the functional moieties responsible during nanoparticle stabilization. The crystalline nature was evaluated by X-ray Diffraction (XRD, Rigaku Miniflex 600), and the size distribution, stability, and charge properties were determined using Dynamic Light Scattering (DLS, Malvern Zetasizer Nano). Scanning Electron Microscopy (SEM, Carl Zeiss, EVO MA18) offered detailed morphological imaging, and the elemental nature was verified by Energy Dispersive X-ray Spectroscopy (EDX). All the dye degradation studies were monitored via UV-visible spectroscopy. The JEM-2100 F Transmission Electron Microscope (TEM) from JEOL, Japan, was utilized for assessing size distribution and analyzing Selected Area Electron Diffraction (SAED) patterns.

### Catalytic reduction of AB 113 dye

To investigate the catalytic reduction of AB 113 dye, experiments were conducted using varying concentrations of the dye solution (10–50 mg/L), with a fixed volume of 3 mL per trial. Each dye solution was added with 100 µL of a freshly prepared 100 mM NaBH_4_ solution in a cuvette to initiate the reduction process. Following this, 100 µL of YFF-AgNPs, colloidal solution was added to the mixture as a catalyst. The reaction progress was monitored using the absorption spectra at periodic intervals to observe spectral changes over time. The concentration of AB 113 in the solution was quantitatively determined using a calibration curve, with the absorption peak measured at 566 nm. The catalytic degradation of AB 113 dye was analyzed using pseudo-first-order kinetic modeling to determine the rate of reaction. The rate constant (k) was calculated using the equation k = ln (C_o_/C_f_) / t, where C_0_ and C_f_ are the initial and residual concentrations of dye at a time ‘t’.

## Results and discussions

### UV-vis spectra analysis

**Fig. 2:**
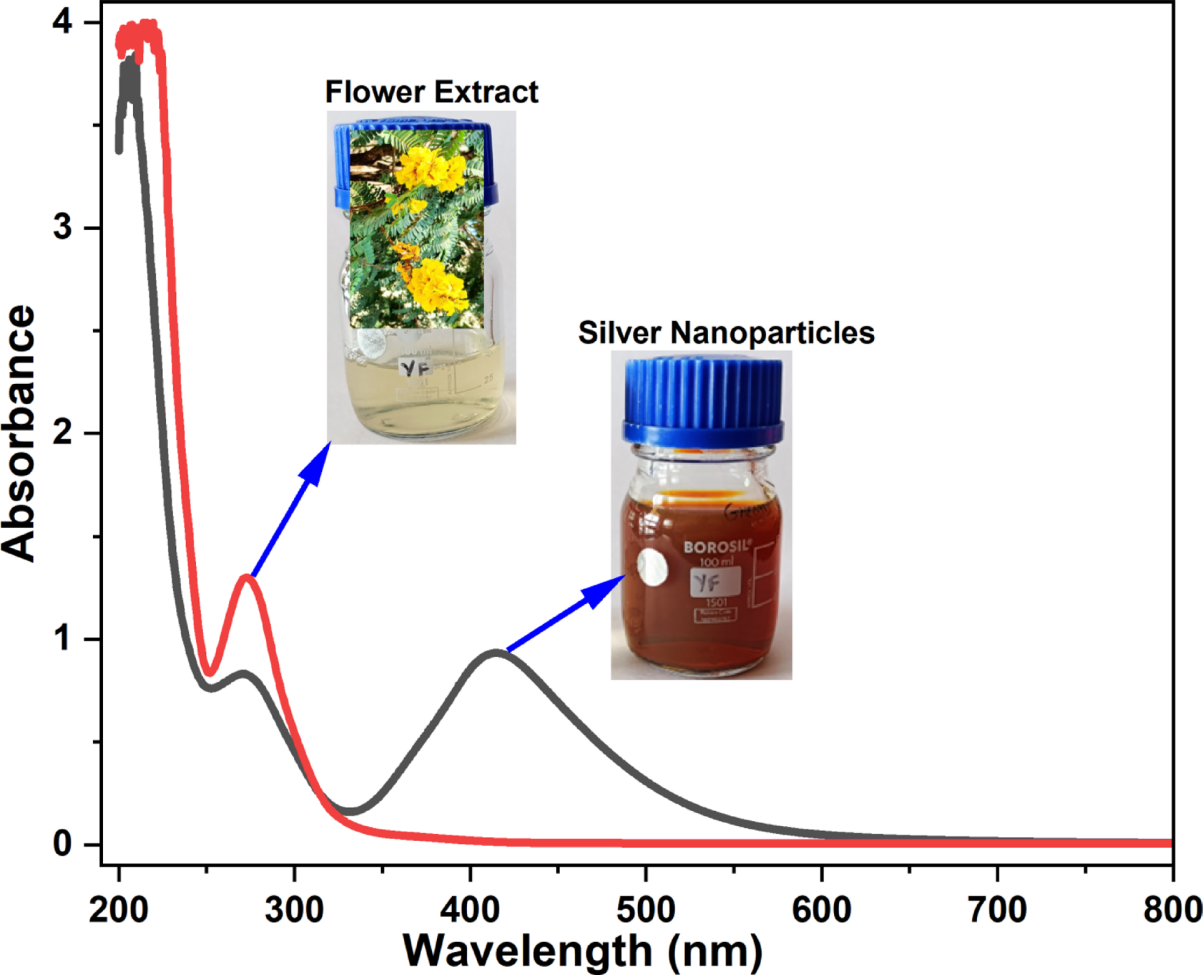
UV-vis spectra of *Peltophorum pterocarpum* flower extract and AgNPs.

The UV-Vis spectra and corresponding images (Fig. 2) of the flower extract and synthesized YFF-AgNPs, reveal key aspects of the nanoparticle synthesis process. As seen in Fig. 2, the flower extract exhibits an absorbance peak at 271 nm, attributable to organic compounds like phenolics or flavonoids, which act as reducing agents that aligns with findings in various studies, such as the preparation of iron oxide NPs using *Cynara cardunculus* leaves extract (269 nm)^14^ and AgNPs with Pathor Kuchi (≈ 265 nm)^15^. Upon adding silver nitrate, the phenolic compounds in the extract reduce Ag^+^ to Ag^0^, as indicated by a new absorbance peak at 416 nm, characteristic of the SPR of AgNPs. This peak is consistent with those reported in studies using *Cucurbita maxima* (414 nm)^16^, and *Amphiroa rigida* (420 nm)^17^ extracts for AgNPs synthesis. The SPR peak confirms the formation of AgNPs, corroborated visually by the solution turning from nearly colorless to brown – a common indicator of AgNP synthesis also observed with *Actiniopteris radiata*^18^ extract. Notably, the intensity of the original 276 nm peak decreases in the spectrum of YFF-AgNPs, compared to the flower extract, suggesting the involvement of these biomolecules during the reduction process. This change supports the role of these organic components not only as reducers but also potentially as stabilizers for the formed nanoparticles. The broad nature of the 416 nm SPR peak suggests a certain degree of polydispersity in the nanoparticle distribution, indicating that while the nanoparticles are somewhat uniform, there is a variation in size^18^. This moderate polydispersity and the involvement of biological molecules in both the reduction and stabilization of YFF-AgNPs, emphasize their dual function in the synthesis process.

### SEM & EDX results

**Fig. 3:**
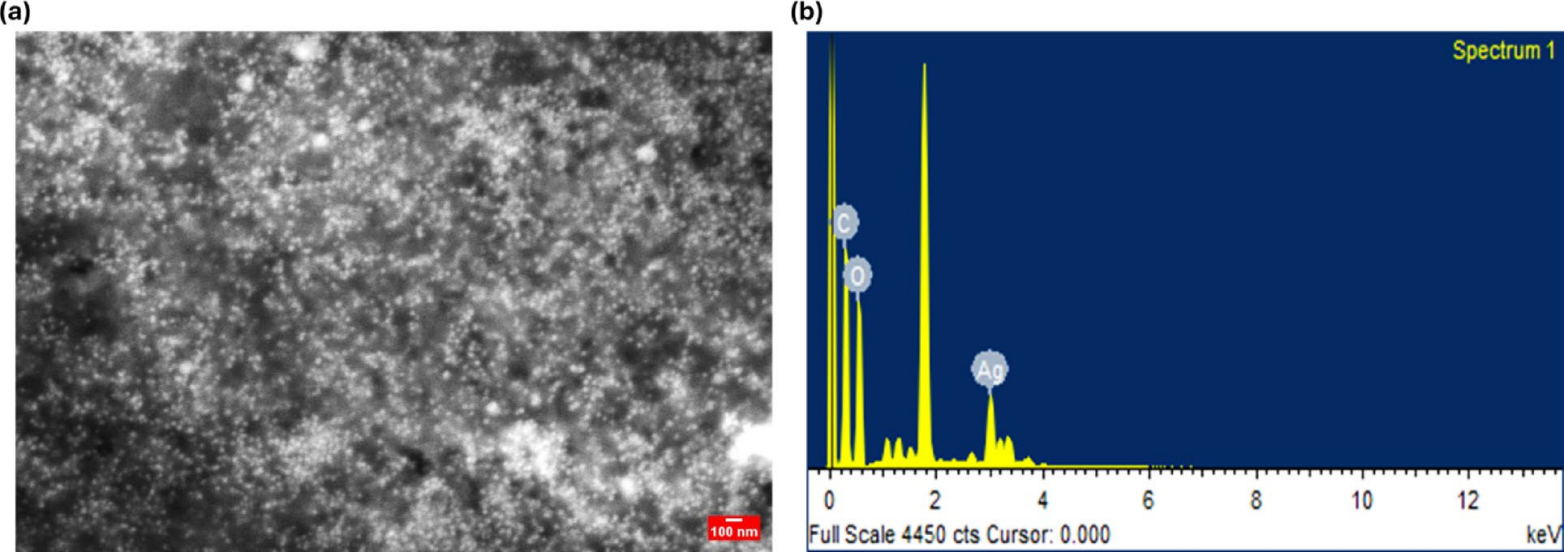
FESEM image (**a**), and EDX spectrum (**b**) of YFF-AgNPs.

This SEM image (Fig. 3a) displays YFF-AgNPs, synthesized using a flower extract, characterized by a uniform, densely packed, and predominantly spherical morphology. Similar shapes are commonly observed in AgNPs synthesized with extracts from *Scrophularia striata*^19^, and *Momordica charantia*^20^ according to recent studies. The distinct, well-separated particles indicate no agglomeration, underscoring the plant extract’s effective stabilizing role in the nanoparticle formation. The EDX spectrum (Fig. 3b) prominently features a peak for Ag at 3 keV, confirming its significant presence. The spectrum also shows signals for carbon at 0.26 keV and oxygen at about 0.53 keV, crucial for the synthesis and stabilization of the nanoparticles, as evidenced in studies like those using *Lythrum salicaria*^21^ and *Abutilon indicum*^22^ extracts for AgNPs synthesis. Notably, the sharp peak around 1.7 keV is identified as Si, likely originating from the glass slide used during sample preparation.

### TEM & SAED patterns

**Fig. 4:**
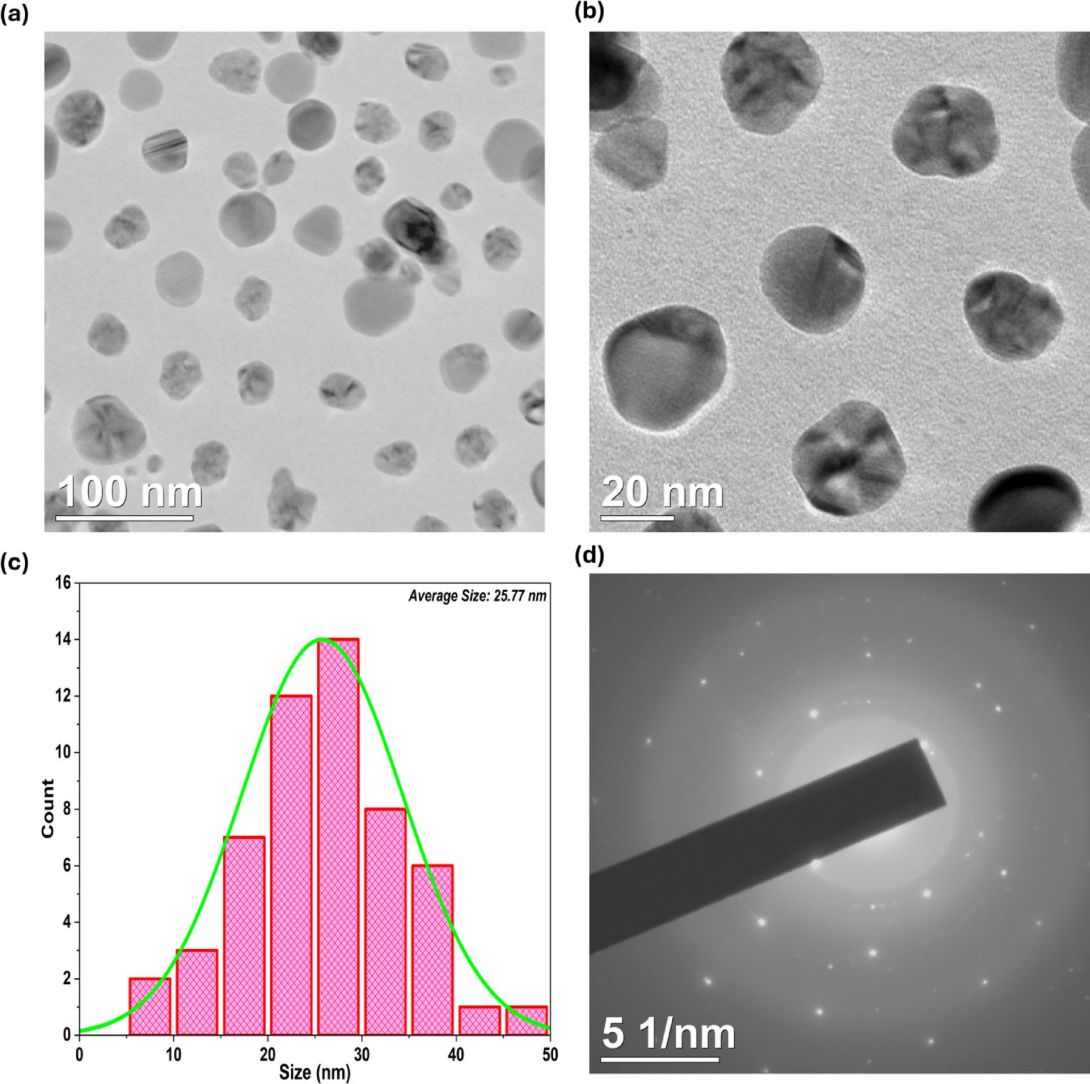
TEM images (**a**) and (**b**), size distribution (**c**), and SAED pattern (**d**) of YFF-AgNPs.

Figure 4a and b showcase TEM images of YFF-AgNPs, synthesized using a flower extract, displayed at different magnifications. These images reveal spherical, monodisperse AgNPs with no agglomeration, indicating that the extract stabilizes and uniformly distributes the nanoparticles. Comparing these results with earlier SEM image confirms the consistent presence of well-defined, discrete nanoparticles. The histogram (Fig. 4c) illustrates the size distribution of the YFF-AgNPs, showing a mean diameter of 25.77 nm, with most particles closely grouped around this value. The distribution peaks between 20 and 30 nm, demonstrating a narrow size range that signifies a high level of uniformity and monodispersity in the synthesis process. In comparison, AgNPs synthesized using *Bauhinia purpurea* flower extract and *Datura metel* exhibited average sizes of 20 nm^23^ and 26 nm^24^ respectively. This underscores the role of the specific plant extract used in influencing the characteristics and dimensions of the nanoparticles. Figure 4d displays the SAED patterns, consisting of bright rings, highlighting the crystalline nature of the YFF-AgNPs. These bright spots and the ring pattern confirm the polycrystalline nature of the nanoparticles^25^, providing further evidence of their structural integrity and uniform crystallinity, essential for their functional applications in various domains.

### XRD results

**Fig. 5:**
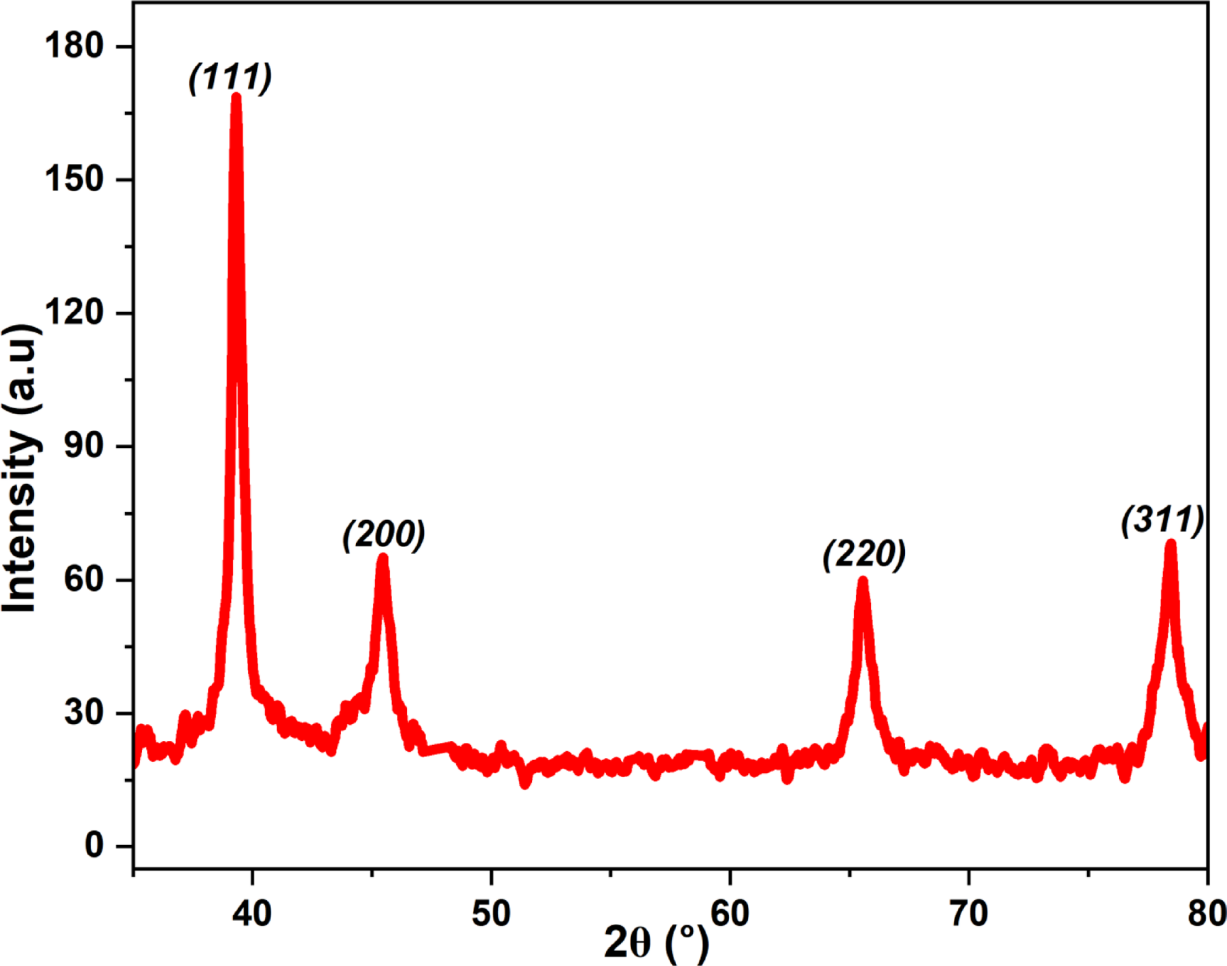
XRD image of YFF-AgNPs.

The XRD analysis of YFF-AgNPs, exhibits distinct peaks at 2θ values of 39.27°, 45.39°, 65.46°, and 78.52°, relating to respective (111), (200), (220), and (311) indices, (Fig. 5). These are indicatory of a face-centered cubic (fcc) arrangement, confirming the crystalline nature as referenced in JCPDS #04-0783^2^. This crystallinity is further evidenced by the bright, concentric rings observed earlier in the SAED pattern, which highlights the polycrystalline nature of the nanoparticles^26^. The calculated d-spacing values of 0.229, 0.199, 0.142, and 0.122 nm for these planes align well with the standard silver lattice parameters, showing slight variations in lattice constants ranging from 0.3970 nm to 0.4037 nm, indicative of minor lattice strains^15^. The most intense peak at (111) suggests that this plane is the most densely packed and the preferred orientation for crystal growth. The mean crystallite diameter calculated as 15.58 nm indicates the presence of multiple crystalline domains within each nanoparticle. This finding aligns with the TEM results showing an overall particle size of 25.77 nm. The difference in particle sizes, as observed between XRD and TEM, is typical, reflecting the aggregation of multiple crystallites and possible amorphous regions at particle boundaries^27^. Such observations are consistent with the literature on fig extract-mediated AgNPs, which reported a mean crystallite diameter of 35.87 nm by XRD and a larger particle size of 45.49 nm by TEM^27^.

### FTIR results

**Fig. 6:**
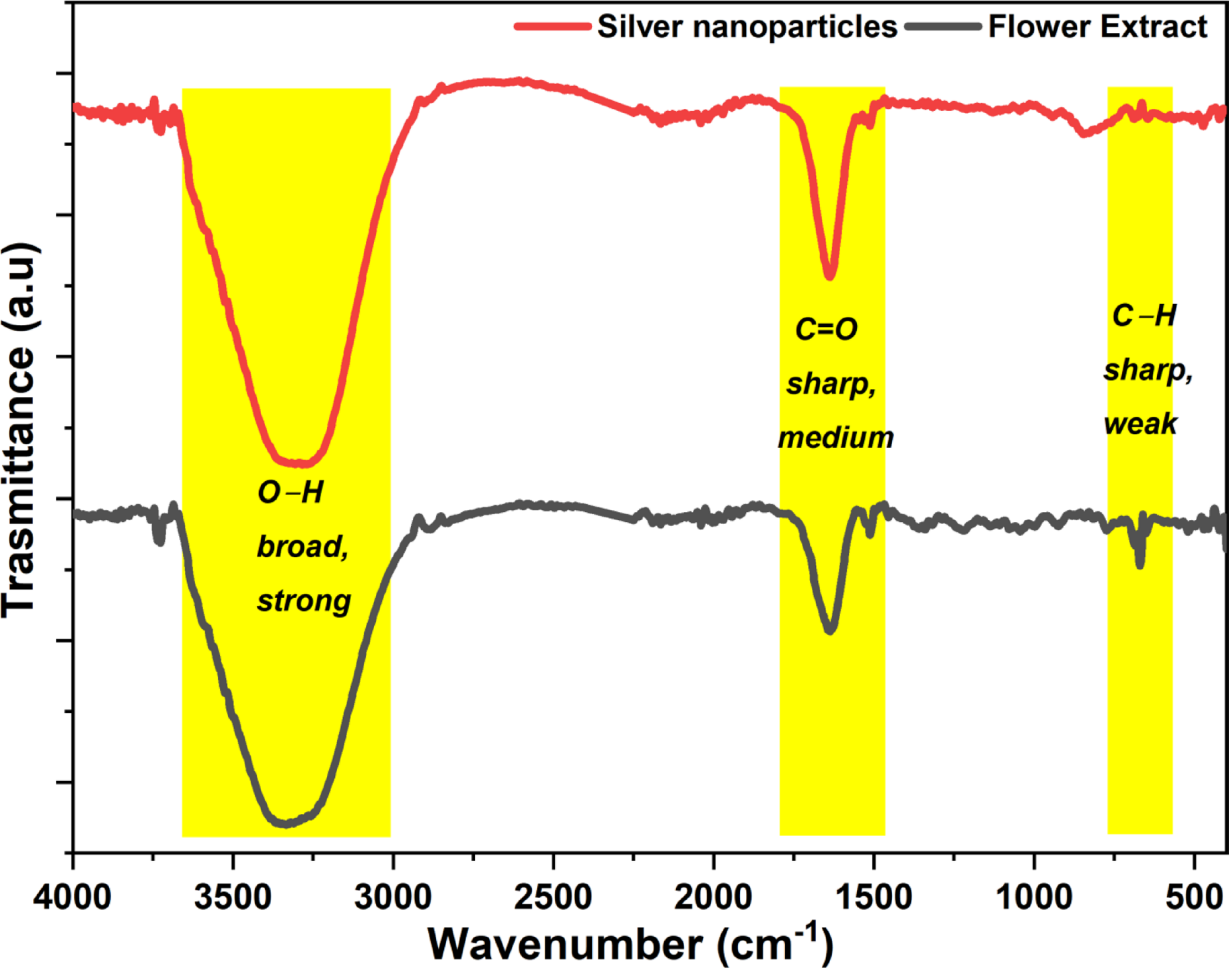
FTIR spectra of *P. pterocarpum* flower extract and AgNPs.

The FTIR spectra of the flower extract and the synthesized YFF-AgNPs, were analyzed to elucidate the functional groups accountable in the AgNPs synthesis process. The flower extract indicates a broad and strong signal (Fig. 6) at approximately 3329 cm^−1^, which is accredited to O-H stretching vibrations typical of hydroxyl moieties^28,29^. Additionally, a sharp and medium signal at 1640 cm^−1^ is observed, corresponding to C=O stretching, possibly from carboxylic acids or amide groups, which suggests their role in reducing and capping the nanoparticles^30^. Notably, in the YFF-AgNPs, spectrum, the 670 cm^−1^ signal associated with the bending vibrations of C–H moieties in aromatic compounds is absent, indicating that these aromatic rings may be involved during synthesis^21^. Moreover, in the YFF-AgNPs, image, the similar broad peak at 3315 cm^−1^ shows reduced intensity, and the peak at 1637 cm^−1^ appears slightly shifted, indicating interactions between these functional groups and the silver surface, which confirms their role in stabilizing the nanoparticles. These observations ally with many reports, including those involving AgNPs synthesized from *Tinospora malabarica* leaf extract^31^ and vegetable waste extract^32^, where similar functional group behaviors and interactions have been observed.

### DLS studies

**Fig. 7:**
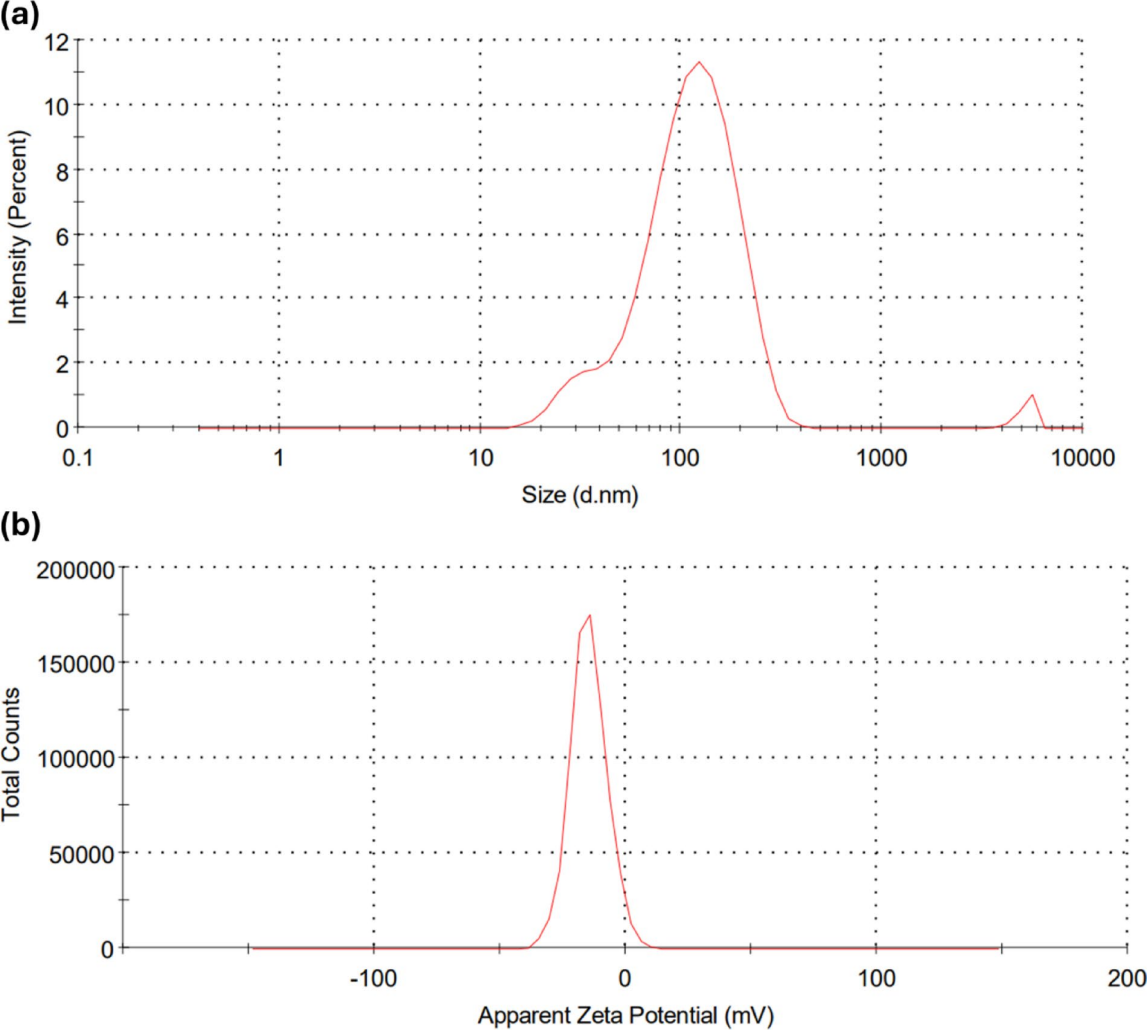
Hydrodynamic size (**a**), and zeta potential (**b**) of YFF-AgNPs.

Dynamic Light Scattering (DLS) studies on the synthesized YFF-AgNPs, provide insights into their size distribution and colloidal stability. The DLS reveals a hydrodynamic diameter of 99.41 nm (Fig. 7a), which is significantly larger than the 25.77 nm observed in TEM. Since the DLS method measures the hydrodynamic size, it generally exceeds the core particle size measured by TEM because of the existence of a solvation layer around the nanoparticles^33^. This phenomenon is consistent with findings from other studies, such as AgNPs synthesized using *A. indicum* leaf extract, which showed a TEM diameter of 20.7 nm and a DLS diameter of 85.64 nm^22^, and AgNPs from marine macroalgae extract with TEM diameter of 11.99 nm and a 50.73 nm DLS diameter^34^. The Polydispersity Index (PDI) of 0.326 for the synthesized YFF-AgNPs, suggests a relatively monodisperse distribution, as PDIs below 0.7 typically indicate a moderately monodisperse system^35^. This finding aligns with literature reports where PDIs of 0.369^22^ and 0.385^36^ were noted for green-synthesized AgNPs. The zeta potential (− 14.7 mV) of the YFF-AgNPs, (Fig. 7b), provides insight into their surface charge and colloidal stability which aligns with the previous studies, such as AgNPs synthesized using *Spondias dulcis* leaf extract and *P. pterocarpum* pod extract, which exhibited zeta potentials of − 15.7 mV^37^ and − 15.8 mV^38^, respectively. This negative value, indicative of the electronegative capping agents from the flower extract coating the nanoparticle surfaces, suggests a moderate level of stability due to electrostatic repulsion between particles^16^. The presence of various biomolecules within the flower extract contributes to this negative zeta potential thereby promoting stabilization.

### Dye degradation studies

**Fig. 8:**
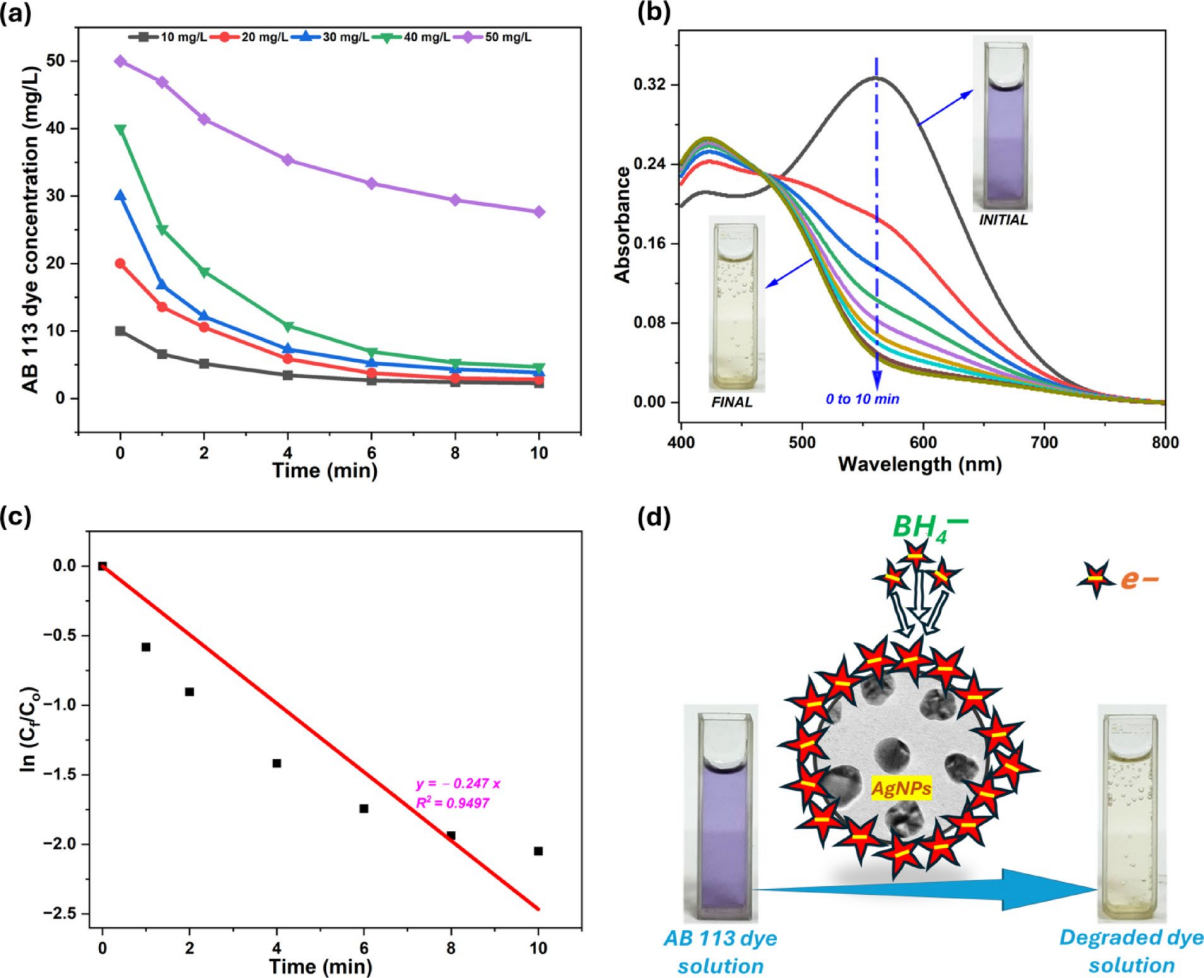
YFF-AgNPs, mediated catalytic degradation of AB 113 dye with NaBH4: Concentration of AB 113 vs. time (**a**), UV-vis spectra of 30 mg/L AB 113 (**b**), Kinetic plot (**c**), and plausible mechanism dye degradation (**d**).

The catalytic degradation of AB 113 dye using YFF-AgNPs, and NaBH_4_ demonstrates remarkable performance (Fig. 8a), particularly notable for its rapid rate of degradation within the first minute across all tested concentrations. This initial phase shows a swift reduction in dye concentration, reducing from 10 to 6.56 mg/L within just one minute, highlighting the catalyst’s immediate and robust activity. As the reaction progresses, the degradation curve begins to flatten, particularly after six min, suggesting a tapering off in the rate of dye reduction. This phenomenon, where the rate of dye reduction slows significantly, is similar to observations made with the degradation of MB dye using *P. pterocarpum*-derived AgNPs, which achieved 82% degradation within six min^11^. The slowing of the reaction could be attributed to the saturation of the AgNPs’ surface with degradation products, which hinders further catalytic activity. As the dye molecules are broken down, the accumulating by-products may occupy active sites, reducing their availability for catalyzing further reactions. This saturation effect limits the efficiency of the AgNPs over time, leading to a noticeable reduction of dye removal as observed for other concentrations as well. Moreover, the removal performance is inversely related to the initial dye concentration. Lower concentrations show a more significant percentage reduction, with the 10 mg/L concentration experiencing approximately 77% reduction within ten min, compared to about a 45% reduction at the 50 mg/L level.

Figure 8b displays the UV-vis spectra of 30 mg/L AB 113 dye degradation, facilitated by the combined use of NaBH_4_ and synthesized YFF-AgNPs. The spectra specifically monitor the reduction in absorbance at 566 nm, a characteristic peak of AB 113 dye. As time progresses, a distinct and consistent reduction in the intensity of this peak is seen, signifying the degradation. By the 10-minute mark, the absorbance at this peak has nearly flattened, indicating substantial degradation of the dye molecules. This change corresponds with the solution’s visible transformation from a vibrant purple to an almost colorless state, as demonstrated in the accompanying images. Such visual and spectroscopic evidence robustly confirms the effective catalytic activity of the YFF-AgNPs, in breaking down the dye, highlighting how AgNPs and NaBH_4_ work synergistically to significantly enhance the degradation process. The observed degradation kinetics adhere to a pseudo-first-order model, indicating that the degradation is primarily dependent on the dye concentration. Figure 8c illustrates a strong linear relationship between ln (C_f_/C_0_) and t for the reduction of AB 113 dye with a coefficient of determination (R^2^) of 0.9497. This high R^2^ value corroborates a very good fit for the linear model, reinforcing the assumption that the reaction adheres to pseudo-first-order model. From the slope of this linear plot, the rate constant (k) for the dye reduction was estimated to be 0.247 min^−1^ which quantifies the efficiency of the dye degradation process, showing a rapid conversion rate.

The mechanism of AB 113 dye reduction, as illustrated in Fig. 8d, vividly demonstrates the critical role of YFF-AgNPs, in facilitating the electron transfer process essential for the degradation of the dye. This process occurs when NaBH_4_ serves as a source of electrons, which are transferred to the surface of the YFF-AgNPs, effectively enhancing their catalytic activity. In this reduction reaction, NaBH_4_ donates electrons, which accumulate on the AgNPs’ expansive surface area^39^. This surface not only supports the adsorption of AB 113 dye molecules but also acts as a robust substrate facilitating the electron transfer reactions. The presence of YFF-AgNPs, possessing an electrochemical potential that mediates between the BH4^−^ ions and the dye molecules, significantly eases the transfer of electrons^10^. As a result, the dye molecules adsorbed on the YFF-AgNPs, surface accept electrons, undergo reduction, and eventually dissociate from the surface in a reduced and colorless form. The transformation of the dye solution from a vibrant purple to a nearly colorless state, as depicted in the accompanying images, provides a compelling visual confirmation of the YFF-AgNPs, catalytic prowess in real-time application.

## Conclusions

The study productively prepared AgNPs using an aqueous extract of *P. pterocarpum* flowers, which acted as both a reducing and stabilizing agent. Morphological assessments through SEM and TEM showed that the nanomaterials were consistently spherical and uniformly distributed. The chemical composition and crystalline structure were validated by EDX and SAED, respectively, affirming the presence of elemental silver and a polycrystalline configuration. Further structural insights were provided by XRD, showing a crystallite size well-matched with theoretical models, and FTIR spectroscopy identified hydroxyl and carboxyl groups as key players in the reduction and stabilization. The process yielded nanomaterials with a high degree of monodispersity and stability, as evidenced by DLS and zeta potential measurements. These nanoparticles exhibited excellent catalytic potential to degrade AB 113 dye, facilitated by sodium borohydride. The kinetic experiments proved that the removal process obeyed the pseudo-first-order model. The use of green chemistry to produce AgNPs not only supports environmental sustainability but also offers a potent method for treating dye-polluted wastewater. Future studies could explore the scalability of the process and further investigate the reusability and long-term stability of the synthesized nanoparticles in various environmental conditions.

## Data Availability

The authors declare that all data supporting the conclusions of this research are contained within the article itself.

## Declarations

### Plant guidelines

The authors affirm that the utilization of plants or plant parts in this research adheres to all applicable international, national, and institutional guidelines.

### Permissions to collect the plants/plant parts

Dr. Gayathri Pai from the Department of Botany at MGM College, Udupi, India, has taxonomically verified and authenticated the tree flowers used in this study. Given that the tree is widely recognized, common, and not under any protection, there was no need for collection permits or licenses. Furthermore, due to its well-known status, no voucher specimen has been deposited in a herbarium.

### Source of the plant used in your study

Details regarding all plant names and their sources are provided in the Methodology section of this document.
